# An Oral Glucose Load Decreases Postprandial Extracellular Vesicles in Obese Adults with and without Prediabetes

**DOI:** 10.3390/nu11030580

**Published:** 2019-03-08

**Authors:** Natalie Z. M. Eichner, Nicole M. Gilbertson, Luca Musante, Sabrina La Salvia, Arthur Weltman, Uta Erdbrügger, Steven K. Malin

**Affiliations:** 1 Department of Kinesiology, University of Virginia, Charlottesville, VA 22903, USA; nze8bz@virginia.edu (N.Z.M.E.); nmg4xk@virginia.edu (N.M.G.); alw2v@virginia.edu (A.W.); 2 Division of Nephrology, University of Virginia, Charlottesville, VA 22903, USA; LM8QD@hscmail.mcc.virginia.edu (L.M.); SL8WM@hscmail.mcc.virginia.edu (S.L.S.); UE2U@hscmail.mcc.virginia.edu (U.E.); 3 Division of Endocrinology and Metabolism, University of Virginia, Charlottesville, VA 22903, USA; 4 Robert M. Berne Cardiovascular Research Center, University of Virginia, Charlottesville, VA 22903, USA

**Keywords:** microparticles, arterial stiffness, insulin sensitivity, type 2 diabetes, obesity

## Abstract

Although extracellular vesicles (EVs) are a novel biomediator of type 2 diabetes (T2D) and cardiovascular disease (CVD), the effects of hyperglycemia on EVs in humans is unknown. We tested the hypothesis that a 75-g oral glucose tolerance test (OGTT) would promote changes in EVs in relation to CVD risk. Twenty-five obese adults (Age: 52.4 ± 3.2 year, BMI: 32.5 ± 1.2 kg/m^2^) were screened for normal glucose tolerance (NGT, *n* = 8) and prediabetes (PD, *n* = 17) using American Diabetes Association criteria (75 g OGTT and/or HbA1c). Body composition (bioelectrical impedance) and fitness (VO_2_peak) were measured. Arterial stiffness (augmentation index; AIx) was measured at 0, 60- and 120-min while insulin, glucose, and free fatty acids were evaluated every 30 min during the OGTT to assess CVD risk. Annexin V positive (AV+) and Annexin V negative (AV−) total EVs, platelet EVs (CD31^+^/CD41^+^; CD41^+^), leukocyte EVs (CD45^+^; CD45^+^/CD41^−^), platelet endothelial cell adhesion molecule (PECAM) (CD31^+^) and endothelial EVs (CD 31^+^/CD41^−^; CD105^+^) were collected at 0 and 120 min. There were no differences in age, BMI, or body fat between NGT and PD (all *P* > 0.63). Total EVs, AV+ CD31^+^ (PECAM), and AV+ CD31^+^/CD41^−^ (endothelial) EVs decreased after the OGTT (*P* ≤ 0.04). Circulating insulin at 2-h correlated with elevated post-prandial AV− CD45^+^ (*r* = 0.48, *P* = 0.04) while arterial stiffness related to reduced total EVs (*r* = −0.49, *P* = 0.03) and AV+ CD41^+^ (platelet) (*r* = −0.52, *P* = 0.02). An oral glucose load lowers post-prandial total, platelet, and endothelial EVs in obese adults with NGT and prediabetes in relation to CVD risk.

## 1. Introduction

Approximately one in three adults in the United States have prediabetes and that number is expected to increase in the upcoming decades [1]. This is concerning since these individuals are at an increased risk for type 2 diabetes (T2D), cardiovascular disease (CVD) and all-cause mortality when compared to individuals with normal glucose tolerance (NGT) [2]. Prediabetes is defined as having either impaired fasting glucose and/or impaired glucose tolerance following a 75-g oral glucose tolerance test (OGTT). This is clinically relevant as postprandial elevations in blood glucose are a better predictor of CVD when compared to fasting glucose alone [3,4]. However, the exact mechanism by which postprandial hyperglycemia confers increased CVD risk remains unclear.

Extracellular vesicles (EVs) have emerged as novel biomediators of T2D and CVD [5,6]. In fact, the addition of endothelial-derived EVs as a parameter to the Framingham risk score significantly improved prediction of future CVD events in high-risk patients [7]. Elevated levels of endothelial-EVs have even been reported in individuals with prediabetes when compared to individuals with NGT [8], suggesting that hyperglycemia is related to EV release. This is consistent with in vitro work suggesting that EVs alter glucose homeostasis and insulin resistance [9,10,11], as well as promote inflammation [12,13], endothelial dysfunction [6,14,15], and vascular stiffening [16,17]. Given these observations are all associated with CVD, it reasons that EVs may be altered during the postprandial state [18]. Interestingly, hyperglycemia per se is considered to be a key factor that impacts EV release, phenotype, and function [19]. In vitro work has shown hyperglycemia to increase endothelial-derived EV (CD 144) formation when compared to controlled glucose conditions, which resulted in greater EV oxidative stress and pro-coagulant activity [20]. In vivo data also support the notion that EV release may be modulated by carbohydrate feeding, as a recent report indicated that endothelial EVs are lowered following carbohydrate restriction in adults with T2D [21]. Taken together, while various stimuli (e.g., lipids and inflammation) have been suggested to impact EV release [18,22], no study has specifically examined the effect of a high glucose load on circulating EVs from endothelium, platelets, and leukocyte-derived origins in people at risk for T2D and/or CVD. This is an important knowledge gap to fill as endothelial, platelet, and leukocyte EVs each have distinct physiological effects [6,23,24]. Therefore, based on previous in vitro work, we tested the hypothesis that total, endothelial-, platelet-, and leukocyte-derived EVs would increase more in response to a 75-g OGTT in individuals with PD compared to those people with NGT and that these elevations in EVs would correlate with CVD risk.

## 2. Materials and Methods

### 2.1. Subjects

Twenty-five adults with obesity were classified as having prediabetes (*n* = 17) or NGT (*n* = 8) using a 2-h 75-g OGTT according to the American Diabetes Association criteria (fasting glucose: 100–125 mg/dL, 2-h glucose 140–199 mg/dL, or HbA1c 5.7–6.4%). Participants were recruited through flyers and advertisements distributed throughout the Charlottesville community as part of two studies conducted within our lab (from 2015 through 2018) as previously described [25]. In total, thirty individuals were recruited for this specific analysis; however, we were unable to collect data for five individuals due to issues with scheduling sample pickup (*n* = 3) and inability to contact after initial enrollment (*n* = 2). Subjects were excluded from participation if physically active (>60 min/week), smoking, or diagnosed with T2D (determined by HbA1c), cardiac dysfunction, cardiopulmonary disorders, cancer (<last 5 years), and/or liver dysfunction. Subjects were also excluded if on medications known to influence insulin sensitivity (e.g., metformin, GLP-1 agonist, etc.) or endothelial function (beta-blockers, angiotensin converting enzyme-inhibitors, etc.). All individuals underwent physical examination that included a resting and exercise 12-lead electrocardiogram, as well as biochemical testing to rule out disease. Upon completion of the entire larger study duration, individuals were compensated $150 for their time. The study was conducted in accordance with the Declaration of Helsinki, and the protocol was approved by the University of Virginia Institutional Review Board (IRB HSR #17822 and #18316) [25].

### 2.2. Metabolic Control

Prior to admission to our Clinical Research Unit (CRU), subjects were instructed to refrain from strenuous exercise or alcohol consumption within 48-h of testing. Subjects were also instructed to refrain from taking any medications or dietary supplements 24-h prior to reporting to the CRU. Subjects were instructed to consume approximately 250 g/day carbohydrate on the day before testing to minimize differences in insulin action. Three-day food logs, including two weekdays and one weekend day, were also used to assess ad-libitum food intake. Participants selected these days and were provided with reference guides that displayed serving sizes of beverages and food. Data were analyzed using ESHA (Version 11.1, Salem, OR, USA) and averaged for analysis.

### 2.3. Body Composition and Aerobic Fitness

Following an approximate 4-h fast, body weight was measured to the nearest 0.01 kg on a digital scale with minimal clothing. Height was measured using a stadiometer for estimations of the body mass index. Body fat and fat-free mass were measured using the InBody 770 Body Composition Analyzer (InBody CO., Cerritos, CA, USA). Waist circumference was obtained 2 cm above the umbilicus twice using a plastic tape measure and averaged. The VO_2_peak was used to assess aerobic fitness and was determined using a continuous progressive exercise test on a cycle ergometer with indirect calorimetry (Carefusion, Vmax CART, Yorba Linda, CA, USA).

### 2.4. Oral Glucose Tolerance Test (OGTT)

Following an approximate 10–12-h fast, subjects reported to the CRU. Subjects were instructed to lay supine undisturbed for at least 5 min to determine resting heart rate (HR) and blood pressure, which was averaged over three measurements for data analysis. Additionally, pulse pressure (defined as systolic-diastolic blood pressure) and mean arterial pressure (((2 × diastolic) + systolic)/3) was calculated. An intravenous line was placed in the antecubital vein for blood sampling. A 75-g OGTT was then performed to assess glucose tolerance and insulin sensitivity. Plasma glucose, insulin, and FFA were measured when subjects were fasted and at 0, 30, 60, 90, and 120 min of the OGTT. Additionally, measures of post-prandial systolic and diastolic blood pressure were obtained at 60 and 120 min. The total area under the curve (tAUC) was calculated using the trapezoidal model. Homeostatic model assessment of insulin resistance (HOMA-IR) was used to assess hepatic insulin resistance as previously described [26]. Adipose insulin resistance was also calculated as the product of plasma insulin and FFA tAUC at 120 min. Whole-body insulin sensitivity, which mostly reflects peripheral glucose metabolism, was assessed by the Matsuda Index [27].

### 2.5. Arterial Stiffness

Fasting and postprandial augmentation index (AIx) was measured by applanation tonometry using the SphygmoCor^®^ system (AtCor Medical, Itasca, IL, USA) at 0, 60, and 120 min of the OGTT while resting quietly semi-supine in a temperature-controlled room. AIx was corrected to a heart rate of 75 bpm using the manufacturer’s software.

### 2.6. EV Preparation and Characterization

Fresh blood when subjects were fasted and at 120 min during the OGTT was drawn using a BD Insyte Autoguard 22G, collected in citrate vacutainers, and processed at room temperature within 120 min of collection. Platelet-poor plasma was obtained by centrifugation (Sovall RC 5B Plus Centrifuge: Rotor SS-34) at 5000× *g* at room temperature for 15 min. An EV pellet was obtained from platelet-poor plasma by a second centrifugation spin (Centrifuge: 524/5424 R-Rotor FA-45-24-11) at 17,000× *g* for 10 min and subsequently washed with AV buffer (1xAVb) (BD Parmingen, San Diego, CA, USA). EV origin was detected using an imaging flow cytometer ImageStream^®^ MKII (Amnis, Seattle, WA) (ISX) using platelet (CD31^+^/CD41^+,^ CD41^+^), leukocyte (CD45^+^/CD41^−^; CD45^+^), platelet endothelial cell adhesion molecule (PECAM) (CD31^+^) and endothelial (CD105^+^, CD31^+^/CD41^−^) protein markers. Annexin V (AV) was used as a membrane dye. Samples and controls were processed as previously described by our group [25,28]. EV counts were also measured by a volumetric method provided by the software of ISX, Tunable resistive pulse sensing and nanosight tracking analysis [25]. Further characterization with cryo-electron microscopy and Western blotting of EV proteins was performed as previously described [25].

### 2.7. Clinical Biochemical Analysis

Plasma glucose samples were analyzed immediately using the YSI 2300 StatPlus Glucose Analyzer system (Yellow Springs, OH, USA). All other samples were centrifuged for 10 min at 4 °C and 3000 rpm, aliquoted, and stored at −80 °C until later analysis. Blood for plasma insulin and FFA was placed in vacutainers containing EDTA and the protease inhibitor aprotinin. Insulin was analyzed using an enzyme-link immunosorbent assay kit (ELISA; Lot #2972715) (Millipore, Billerica, MA, USA) and circulating FFAs were analyzed using an enzymatic colorimetric method assay (Wako Diagnostics, Richmond, VA, USA). Fasted intercellular adhesion molecule (ICAM) and vascular cell adhesion molecule (VCAM) were also measured using ELISA (R&D Systems, Minneapolis, MN, USA).

### 2.8. Statistical Analysis

Data were analyzed using SPSS v24 (Armonk, NY, USA). Normality was assessed using Shapiro–Wilk tests. EV data were log-transformed due to non-normality. Independent, two-tailed *t*-tests were used to determine baseline group differences between NGT and prediabetes. A repeated measures ANOVA was utilized to compare group differences following the OGTT. Given that aerobic fitness was significantly different between NGT and prediabetes, VO_2_peak was used as a covariate. Given the unequal numbers between NGT and prediabetes, we also elected to perform a subgroup analysis based on prediabetes phenotypes. Since no differences were noted (data not shown), all prediabetes data were pooled for comparison. Pearson correlations were used to test associations between EVs and clinical outcomes. Significance was set at *P* < 0.05, and trends were accepted at *P* = 0.05–0.10. Data are reported as mean ± SEM.

## 3. Results

### 3.1. Subject Characteristics

Body weight and body fat percent were similar between individuals with NGT and prediabetes, see Table 1. However, adults with prediabetes had a lower relative VO_2_peak (mL/kg/min) compared to those with NGT (*P* < 0.02). There were no significant group differences in resting HR in those with NGT and prediabetes (74.8 ± 2.1 vs. 70.9 ± 2.3 bpm, *P* = 0.31, respectively), nor in HDL (*P* = 0.71), LDL (*P* = 0.37), total cholesterol (*P* = 0.28) or triglycerides (*P* = 0.44). There were also no significant differences between total calories (*P* = 0.70), carbohydrate (*P* = 0.21), fat (*P* = 0.66), or protein (*P* = 0.99) between NGT and prediabetic adults in ad-libitum diet consumption, see Table 1.

### 3.2. Glucose Tolerance

By design, fasting (*P* = 0.006) as well as 2-h glucose was higher in those with prediabetes compared with NGT (*P* = 0.006), see Table 2. However, HbA1c did not differ between groups (NGT = 5.4 ± 0.09 vs. prediabetes = 5.6 ± 0.07%, *P* = 0.11). As expected, glucose increased in response to the 75-g OGTT in both NGT and those with prediabetes (time effect: *P* = 0.005; gxt: *P* = 0.13). However, adults with prediabetes had higher tAUC_120_ glucose when compared NGT (*P* = 0.05), see Table 2.

### 3.3. Insulin Sensitivity and FFA

People with prediabetes had higher fasting insulin (*P* = 0.03) than those with NGT. Baseline FFA was not different between groups (*P* = 0.92). Insulin significantly increased in response to the OGTT at 120 min, while FFA decreased (both *P* < 0.001). Whole-body insulin sensitivity was higher in NGT versus those with prediabetes (*P* = 0.02) and both HOMA-IR and adipose insulin resistance were significantly lower in NGT versus prediabetics (both *P* < 0.03), see Table 2.

### 3.4. Arterial Stiffness, Blood Pressure, and Inflammation

There was no baseline AIx difference between groups. However, while not statistically significant, AIx at 2 h tended to decrease in people with NGT and prediabetes after the 75-g glucose load (time effect: *P* = 0.06), as shown in Table 3. There was also no difference for baseline blood pressure between individuals with NGT and prediabetes, see Table 3. However, systolic blood pressure (SBP), diastolic blood pressure (DBP) and mean arterial pressure (MAP) were significantly elevated at 120 min following administration of the OGTT (time effect: all *P* < 0.02), while PP was reduced (*P* < 0.001) in both NGT and prediabetes. Although ICAM was significantly elevated in adults with prediabetes compared to those with NGT (178.4 ± 12.6 vs. 226.5 ± 12.7 ng/mL, *P* = 0.04), VCAM was similar between the two groups (592.6 ± 41.5 vs. 697.0 ± 50.7 ng/mL, *P* = 0.25).

### 3.5. Extracellular Vesicles

**Figure 1:**
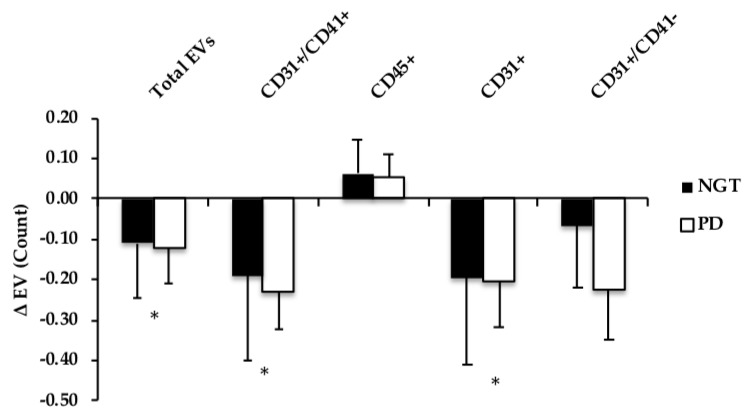
Comparison of changes (Δ; fed-fasted) in Annexin V+ (AV+) EV subtype count following an oral glucose tolerance test (OGTT) in NGT vs. prediabetes (PD). EV data were log-transformed. * Time effect, *P* ≤ 0.05.

**Figure 2:**
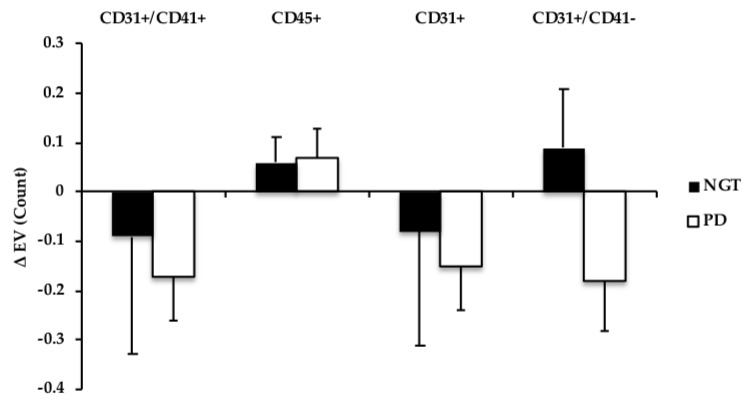
Comparison of changes (Δ; fed-fasted) in Annexin V− (AV−) EV subtype count following an OGTT in NGT vs. prediabetes (PD). EV data were log-transformed. CD31+/CD41+ and CD31+ tended to decrease; Time effect, *P* ≤ 0.10.

Fasting EVs were not different at the baseline between adults with NGT and prediabetes. Total EVs, EV AV+ CD31^+^ (PECAM), as well as platelet EVs AV+ CD41^+^, and endothelial AV+ CD31^+^/CD41^−^, all decreased at 120 min during the OGTT (time effect: all *P* ≤ 0.04), see Figure 1. AV− CD31^+^ (PECAM) and AV− CD 31^+^/CD41^−^ (endothelial) also tended to be lowered following the glucose load, but did not reach statistical significance (time effect: *P* ≤ 0.10), see Figure 2. Leukocyte-derived AV+ EVs and endothelial EV CD105 were not different from baseline at 120 min during the OGTT (gxt effect: *P* = 0.14, 0.92, respectively). When cardiorespiratory fitness was included as a covariate in the ANOVA model, the postprandial responses for total EV (*P* = 0.11), AV+ PECAM CD31^+^ (*P* = 0.14), and platelet AV+ CD31^+^/CD41^+^ (*P* = 0.28) EVs were mitigated.

### 3.6. Correlations

**Figure 3:**
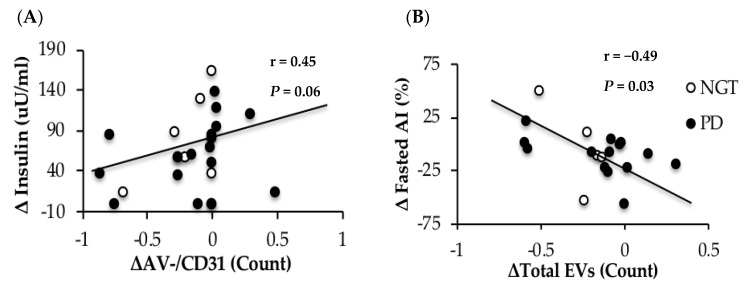
Correlation between changes (Δ; fed-fasted) in EVs, insulin (**A**) and arterial stiffness, as assessed by augmentation index (**B**) EV data were log-transformed.

Elevations in postprandial insulin correlated with larger increases in AV− CD31^+^ EVs following a 75-g OGTT in obese adults with and without prediabetes, see Figure 3A. Although not statistically significant, we report trends for increased circulating insulin at 120 min correlating with elevated post-prandial AV+ CD105 (*r* = 0.45, *P* = 0.06) and AV− CD45^+^ (*r* = 0.48, *P* = 0.04). Additionally, those with greater whole-body insulin sensitivity saw declines in leukocyte-derived AV− CD45^+^ (*r* = −0.45, *P* = 0.06) and AV− CD41^+^/CD45^+^ (*r* = −0.47, *P* = 0.05) in response to the glucose load. Moreover, postprandial changes in total EVs (*r* = 0.46, *P* = 0.07), AV+ CD45^+^ (*r* = 0.46, *P* = 0.05) and AV+ CD105 (*r* = 0.55, *P* = 0.02) were all positively related to adipose insulin resistance. Increased HOMA-IR was associated with increased baseline leukocyte AV+ CD45^+^ EVs (*r* = 0.38, *P* = 0.08). Changes in AIx were also associated with reduced total EVs following the OGTT (*r* = −0.49, *P* = 0.03) (Figure 3B) and AV+CD41^+^(platelet) (*r* = −0.52, *P* = 0.02). Fasted LDL (*r* = 0.49, *P* = 0.07) and triglycerides (*r* = 0.57, *P* = 0.03) were related to baseline levels of AV+/−CD45^+^ and higher baseline FFA (*r* = 0.42, *P* = 0.10) and cholesterol (*r* = 0.56, *P* = 0.03) were related to elevated post-prandial AV+CD45^+^. Aerobic fitness tended to correlate with lower insulin levels at 120 min of the OGTT (*r* = −0.41, *P* = 0.07) as well as whole-body insulin sensitivity (*r* = 0.35, *P* = 0.10).

## 4. Discussion

In contrast to our hypothesis, the primary observation of this study is that a single oral glucose load lowers total, platelet, and PECAM EVs in obese adults with both NGT and prediabetes. Although the reduction in EVs in response to an oral glucose load was associated with reduced insulin sensitivity and increased arterial stiffness, when VO_2_peak was included as a covariate, the effect of an oral glucose load on EVs was weakened. Overall, our results support the notion that EVs may relate to CVD risk in adults with obesity, independent of glucose status.

To date, there are no human studies examining the acute effect of carbohydrate feeding on EVs. Prior work by Francois et al. suggested that carbohydrate restriction for four days in T2D lowered EVs, although this did not relate to improved endothelial function [21]. However, it is possible that these changes were also related to a significant decrease in the total calories consumed, as individuals were given three meals equating to 500 kcal/meal over four days. Similarly, Wekesa et al. also report significant decreases in the endothelial EV CD31^+^/CD41^−^ following 24 weeks of a low-carbohydrate diet in obese women [29]. In contrast, individuals in the present study were instructed to make no changes to their ad-libitum diet three days prior to the collection of EVs, and there were no differences in the ad-libitum diet between NGT and adults with prediabetes, suggesting the present results are independent of energy or carbohydrate restriction. Notwithstanding differences in glucose versus total carbohydrate differences between the current and prior studies [21,29], our data are also in contrast to previous literature that reported a significant increase in endothelial EVs in response to a single high glucose load in vitro [20]. It is important to note, however, that this prior study treated human umbilical vein endothelial cells (HUVECs) with a glucose load equivalent to approximately 450 mg/dL. In the present study, adults with prediabetes reached an average of only 136.8 mg/dL at 2-h following administration of the 75-g OGTT. Taken together, our findings identify the need for further work on understanding the impact of dietary intake on EVs to improve T2D and CVD risk management.

The reductions in EV following a glucose load in adults with obesity may be explained by different potential mechanisms. We previously showed that aerobic fitness is associated with lower fasting platelet and endothelial EVs [25]. Therefore, it would be reasonable to expect that the VO_2_peak may, in part, influence the postprandial EV response as well. Indeed, our results support this notion, as accounting for differences in aerobic fitness weakened the effect of an oral glucose load on decreasing total EV (*P* = 0.11), PECAM CD31^+^ (*P* = 0.14), and platelet CD31^+^/CD41^+^ (*P* = 0.28). The reason fitness attenuates the effect of the OGTT on EVs is beyond the scope of this study, but improved aerobic fitness is related to insulin sensitivity [30] and we report in the present study that VO_2_peak tended to correlate with insulin concentrations. Interestingly, recent work demonstrates that EVs derived from macrophages [9] and adipocytes [31] reduce insulin-stimulated glucose uptake in the liver [10] and skeletal muscle [11]. Moreover, macrophage-derived EVs interfere with GLUT-4 translocation in human adipocytes by decreasing Akt-phosphorylation [9]. The reason for this decreased insulin signaling was proposed to be mediated by NFkB, suggesting that inflammation plays a role in EV-induced insulin resistance [9]. The results herein are in line with previous in vitro work, as we report that platelet-derived (AV− CD31^+^/CD41^+^) EVs and the PECAM EV AV− CD31^+^ are significantly related to the postprandial insulin response during an OGTT, see Figure 3A, as well as higher total, leukocyte (AV+ CD45^+^), and endothelial EVs (AV+ CD105) correlate with adipose insulin resistance, which is consistent with work showing that insulin resistance increases EVs secretion and alters circulating leukocyte function [32]. Thus, our findings suggest for the first time that fitness-related insulin sensitivity may modulate EV responses to an oral glucose load in obese adults with or without prediabetes.

Another factor by which the postprandial response may relate to EVs is through vascular function. Circulating endothelial EVs are thought to play a key physiologic role in vascular physiology [6,22] and are associated with reduced endothelium-dependent relaxation [14], lower flow-mediated dilation [17], increased pulse wave velocity (PWV) [17], and higher carotid intima-media thickness [16]. In response to this high glucose load, AIx, an index of systemic arterial stiffness, was lowered at 120 min of the OGTT. This is consistent with prior work [33], suggesting that feeding lowers arterial stiffness for increased nutrient delivery. Interestingly, reductions in EVs in our study were associated with preservation/elevations in arterial stiffness, see Figure 3B. Our finding suggests that total postprandial EVs may relate to impaired vascular function following a high glucose load. It is possible that this decrease in postprandial EVs relates to less ability for the blood vessel to vasodilate from nitric oxide, as previous work has shown circulating EVs to carry endothelial nitric oxide synthase (eNOS) [34].

Vascular inflammation may also modulate EV release in the present study [6]. Despite higher levels of ICAM in adults with prediabetes compared with NGT, we report no difference in EV response to the glucose challenge. Moreover, VCAM nor ICAM were related to baseline or postprandial EV responses. It is important to note, however, that we did not characterize other markers of inflammation, such as IL-6 or CRP, that are also known to modulate EV release and it remains possible these or other inflammatory mechanisms are at play [35]. In either case, it seems from our data that vascular inflammation is not a primary driver of EV levels in adults with obesity.

In this present study, leukocyte-derived EVs were not lowered in response to the OGTT as were PECAM (CD31^+^) and platelet-derived (CD31^+^/CD41^+^) EVs, see Figure 1 and Figure 2. This raises the possibility that leukocyte EVs may be more intimately related to another stimulus, such as postprandial dyslipidemia, compared to endothelial- or platelet-derived EVs. Indeed, as our group [25] and others [23] have shown leukocyte EVs are inversely related to HDL concentrations, in addition to participating in atherosclerosis progression [23]. Herein, we expand this prior work and report significant relationships between fasted LDL, triglycerides, and leukocyte-derived EV CD45^+^ in this present cohort. Additionally, previous in vitro data have suggested lipotoxicity modulates the release of EVs [36]. We now add to the literature by showing that fasting FFA and cholesterol concentrations, as well as hepatic and adipose insulin resistance, also relate to postprandial AV+ CD45^+^ responses. Collectively, these data suggest that circulating lipids, not glucose, may be an important modulator of leukocyte-derived EVs.

This study has a relatively small sample size and should be considered preliminary in nature. Further work is required to understand if individuals across sex/race respond similarly. Another consideration is that despite individuals with prediabetes having elevated post-prandial glucose and insulin concentrations versus those with NGT, HbA1c was not statistically different between the two groups (*P* = 0.11). This may help explain why we did not see a differential EV response based on glucose status. We attempted to examine people with NGT vs. people with elevated fasting glucose and those with impaired glucose tolerance (n = 7) within this data set, and still, EVs decreased post-OGTT. Nonetheless, future work should consider examining the impact of dietary glucose load on EV responses in adults with either more severe forms of prediabetes and/or T2D compared to individuals with NGT. Moreover, we tested the effects of a single oral glucose load on EVs, and it is possible that a high-fat or mixed-meal would elicit differential EV responses. However, use of the OGTT provides clinical relevance and proof of concept for the effect of a glucose bolus relative to in vitro data. In addition, we only measured EVs at 120 min post-oral glucose load. While this time-point corresponds to the 120 min glucose criteria used to predict future CVD risk, it remains possible that EVs may respond to an acute nutrient load in a mono- or biphasic nature, as has been shown for circulating glucose [37]. Interestingly, we report significant effects of hyperglycemia on AV+ EVs, with only some trends on AV− EVs. Previously, we have shown AV− EVs to be elevated in adults with lower cardiorespiratory fitness [25]. Although it is beyond the scope of the present study to determine why we do not see significant effects of hyperglycemia on AV− EVs, we speculate that this may be due to difference in stimuli, as Connor et al. reported the proportion of EVs that bound Annexin was dependent upon the agonist of EV release [24]. Taken together, additional work is needed to better understand the differing functions of AV+/− EVs, as our data suggests they may differentially respond to varying stimuli (i.e., fitness, feeding). Finally, associations do not equal causation and future work is needed to test how nutrients impact in vivo EV cargo (e.g., mRNA, proteins, etc.) and function [38] to illuminate roles these cells have on human physiology and disease risk.

## 5. Conclusions

In conclusion, a single oral glucose load lowers total, platelet, and PECAM EVs in obese adults with NGT and prediabetes. These findings may have clinical relevance, as the reductions in EVs were associated with insulin sensitivity and arterial stiffness. However, aerobic fitness may, in part, mediate the regulation of postprandial response of these EVs. Therefore, future research is warranted to examine whether training status and habitual dietary carbohydrate intake impact EV subtypes in a cell-specific manner to reduce T2D and CVD risk for optimization of disease care and management.

## Figures and Tables

**Table 1:** Baseline demographics.

	NGT	Prediabetes	*P*-Value
N (M/F)	8 (1/7)	17 (4/13)	-
Age (year)	50.1 ± 5.1	53.5 ± 4.1	0.63
Body weight (kg)	90.3 ± 2.9	98.1 ± 3.0	0.34
Body Mass Index (kg/m^2^)	33.2 ± 1.4	32.1 ± 1.6	0.67
Body Fat (%)	42.3 ± 2.9	44.1 ± 5.3	0.64
Fat Free Mass (kg)	53.9 ± 1.9	53.7 ± 2.9	0.95
VO_2_peak (L/min)	2.1 ± 0.2	1.8 ± 0.1	0.10
VO_2_peak (mL/kg/min)	23.4 ± 1.8	18.6 ± 0.9 *	0.01
HDL (mg/dL)	49.7 ± 3.0	52.8 ± 5.9	0.71
LDL (mg/dL)	111.3 ± 7.0	131.7 ± 15.8	0.37
Triglycerides (mg/dL)	103.0 ± 22.2	136.7 ± 28.8	0.44
Cholesterol (mg/dL)	178.0 ± 8.4	207.1 ± 18.4	0.28
Calories	2381.7 ± 263.0	2261.7 ± 263.0	0.70
CHO (g)	291.4 ± 29.4	244.1 ± 21.2	0.21
Sugar (g)	107.1 ± 17.2	93.7 ± 8.7	0.45
Total Fiber (g)	23.6 ± 2.8	19.0 ± 1.3	0.10
Fat (g)	97.7 ± 14.6	91.4 ± 26.5	0.66
Protein (g)	86.4 ± 10.9	86.5 ± 7.2	0.99

Data are mean ± SEM. Normal glucose tolerance (NGT); high density lipoprotein (HDL); low density lipoprotein (LDL); total carbohydrate (CHO). Significant differences (* *P* ≤ 0.05). Differences in fitness accounted for in the ANOVA model mitigated the postprandial response of extracellular vesicles (EVs).

**Table 2:** Glucose regulation in adults with normal glucose tolerance (NGT) and prediabetes.

	NGT (*n* = 8)	Prediabetes (*n* = 17)	*P*-Value
Fasting PG (mg/dL)	95.2 ± 1.3	104.9 ± 2.1 *	0.006
120 min PG (mg/dL)	105.7 ± 9.2	136.8 ± 9.2	0.63
PG tAUC_120_ (mg/dL·min)	15,046.6 ± 550.0	17,593.8 ± 1089.3 *	0.05
Fasting Insulin (μU/mL)	13.8 ± 2.2	12.8 ± 2.4 *	0.03
120 min Insulin (μU/mL)	55.3 ± 11.3	107.0 ± 11.8 *	0.01
Insulin tAUC_120_ (μU/mL·min)	53.9 ± 1.9	53.7 ± 2.9	0.20
Fasting FFA (mEq/L)	0.52 ± 0.07	0.53 ± 0.03	0.92
120 min FFA (mEq/L)	0.12 ± 0.05	0.22 ± 0.05	0.20
FFA tAUC_120_ (mEq/L·min)	31.3 ± 6.4	45.7 ± 6.7	0.20
HOMA-IR	2.4 ± 0.4	4.5 ± 0.7 *	0.02
Adipose insulin resistance	19.2 ± 3.9	35.9 ± 6.0 *	0.03
Whole-Body Insulin Sensitivity	4.0 ± 1.0	2.1 ± 0.3 *	0.02
HbA1c (%)	5.4 ± 0.09	5.6 ± 0.07	0.11

Data are mean mean ± SEM. Significant differences (* *P* ≤ 0.05). Plasma glucose (PG); total area under the curve (tAUC); free fatty acid (FFA); Homeostatic model of insulin resistance (HOMA-IR).

**Table 3:** Cardiovascular disease (CVD) risk factors in adults with normal glucose tolerance (NGT) and prediabetes.

	NGT (*n* = 8)		Prediabetes (*n* = 17)		ANOVA (*P*-Value)	
	0 min	120 min	0 min	120 min	Time	GxT
Systolic BP (mmHg)	125.0 ± 3.7	134.1 ± 6.7 *	129.2 ± 4.8	137.6 ± 4.4 *	0.02	0.88
Diastolic BP (mmHg)	71.9 ± 4.1	77.1 ± 4.6 *	71.3 ± 3.3	81.7 ± 2.7 *	0.009	0.35
MAP (mmHg)	87.2 ± 3.5	99.4 ± 5.8 *	91.4 ± 3.7	99.4 ± 3.3 *	0.001	0.42
Pulse Pressure (mmHg)	51.6 ± 2.8	41.9 ± 3.2 *	57.9 ± 2.3	42.3 ± 2.0 *	<0.001	0.22
RHR (bpm)	66.8 ± 2.0	66.2 ± 2.0 *	66.6 ± 2.9	63.4 ± 1.8 *	0.02	0.44
AIx (%)	32.1 ± 7.8	17.3 ± 9.5	23.4 ± 2.1	20.3 ± 2.7	0.06	0.21

Data are mean ± SEM. No significant baseline differences existed between groups for any outcome. Blood pressure (BP); mean arterial pressure (MAP); resting heart rate (RHR); augmentation index (AIx). Time Effect, * *P* ≤ 0.05.
